# Intelligent Human Operator Mental Fatigue Assessment Method Based on Gaze Movement Monitoring

**DOI:** 10.3390/s24216805

**Published:** 2024-10-23

**Authors:** Alexey Kashevnik, Svetlana Kovalenko, Anton Mamonov, Batol Hamoud, Aleksandr Bulygin, Vladislav Kuznetsov, Irina Shoshina, Ivan Brak, Gleb Kiselev

**Affiliations:** 1 St. Petersburg Federal Research Center of the Russian Academy of Sciences (SPC RAS), St. Petersburg 199178, Russia; hamoud.b@iias.spb.su (B.H.); alexandr_bulygin@mail.ru (A.B.); 2 Laboratory for Cognitive Psychology of Digital Interface Users, HSE University, Moscow 101000, Russia; sv.d.kovalenko@gmail.com; 3 Faculty of Physics and Mathematics and Natural Sciences, Peoples’ Friendship University of Russia, Moscow 117198, Russia; anton.mamonov.golohvastogo@mail.ru (A.M.); i.v.brak@gmail.com (I.B.); kiselev@isa.ru (G.K.); 4 Digital Education Department, Moscow State University of Psychology and Pedagogy, Moscow 127051, Russia; 5 Federal Research Center “Computer Science and Control” of Russian Academy of Sciences (FRC CSC RAS), Moscow 119333, Russia; ivladnet@ya.ru; 6 Institute for Cognitive Research, Saint Petersburg State University, St. Petersburg 199034, Russia; shoshinaii@mail.ru; 7 Faculty of Information Technologies, Novosibirsk State University, Novosibirsk 630090, Russia

**Keywords:** mental fatigue detection, eye-tracking, machine learning

## Abstract

Modern mental fatigue detection methods include many parameters for evaluation. For example, many researchers use human subjective evaluation or driving parameters to assess this human condition. Development of a method for detecting the functional state of mental fatigue is an extremely important task. Despite the fact that human operator support systems are becoming more and more widespread, at the moment there is no open-source solution that can monitor this human state based on eye movement monitoring in real time and with high accuracy. Such a method allows the prevention of a large number of potential hazardous situations and accidents in critical industries (nuclear stations, transport systems, and air traffic control). This paper describes the developed method for mental fatigue detection based on human eye movements. We based our research on a developed earlier dataset that included captured eye-tracking data of human operators that implemented different tasks during the day. In the scope of the developed method, we propose a technique for the determination of the most relevant gaze characteristics for mental fatigue state detection. The developed method includes the following machine learning techniques for human state classification: random forest, decision tree, and multilayered perceptron. The experimental results showed that the most relevant characteristics are as follows: average velocity within the fixation area; average curvature of the gaze trajectory; minimum curvature of the gaze trajectory; minimum saccade length; percentage of fixations shorter than 150 ms; and proportion of time spent in fixations shorter than 150 milliseconds. The processing of eye movement data using the proposed method is performed in real time, with the maximum accuracy (0.85) and F1-score (0.80) reached using the random forest method.

## 1. Introduction

Nowadays, the severity of the impact of human operator mental fatigue during the implementation of working tasks is currently underestimated. According to the National Highway Traffic Safety Administration (NHTSA), drowsy driving (a symptom of mental fatigue) was reportedly involved in 1.8% of fatal crashes from 2017 to 2021 [1]. According to the New Zealand Ministry of Transport, “In 2022 there were 34 fatal crashes, 80 serious injury crashes, and 460 minor injury crashes where driver fatigue was a contributing factor” [2].

Although mental fatigue has traditionally been regarded as a parameter affecting cognitive performance, in recent years scientists have found increasing evidence that prolonged performance of a monotonous task also affects the human physiological state [3]. Ways of determining mental fatigue include the analysis of different types of signals. Thus, the most common physiological characteristics that reflect this functional state are heart rate variability, electrical brain activity, skin galvanic response, respiratory rate, and face and eye movements.

The authors point out that the existing methods of fatigue detection in drivers insufficiently take into account the influence of mental fatigue on the human condition [4]. In recent years, due to the development of machine learning techniques, the accuracy and speed of detection algorithms have reached a new level. The authors of the article, on recognition of the state of operators, developed an LSTM for analyzing eye movements. As a result of the experiments conducted, the effectiveness of its development was confirmed [5]. For example, in a study of fatigue detection in excavator drivers, the authors pointed out that the appearance of subjective fatigue is accompanied by an increase in reaction time and number of errors. On the side of changes in oculomotor characteristics, the authors emphasize the change in the distribution of fixation points, which means that it becomes difficult for operators to see the surroundings clearly [6].

This paper presents a method for human operator gaze monitoring to identify characteristics that show an increase in mental fatigue. We use our previously developed dataset [7], that includes longitude gaze data recordings of human operators, to identify the gaze characteristics most related to mental fatigue, as well as propose a classifier that detects a fatigue state with high accuracy. We use correlation analysis of all gaze characteristics with different tests: Landolt rings, inner session dynamics, choice reaction times, and the visual analogue scale. Then, experts analyze this correlation and identify the seven gaze characteristics most related to the fatigue state. After that, we prove this choice using experimental results. Therefore, the scientific novelty of the paper includes the following:

- A novel technique for identification of the gaze characteristics that are most related to a mental fatigue state;
- A list of seven gaze characteristics that show the best results for mental fatigue classification in the considered dataset;
- A classifier that allows the assessment of a mental fatigue state based on gaze movement data.

We have developed an open-source Python library that is available on GitHub (https://github.com/AI-group-72/FAEyeTON, accessed on 21 October 2024). The library is published under the GNU Lesser General Public License, version 2.1, and is also available at the PyPI repository, named eyetrackfatigue. It can be easily integrated into target business software, with key functions (we show the examples in test files, displayed in the repository). The library uses pandas to work with data files. The ML models used in the library are based on the scikit-learn library, and can be easily expanded. Additionally, the library has an option for running as a standalone application, with a PyQt-based user interface, for demonstrational purposes.

The rest of the paper is organized as follows. Section 2 includes an overview of the research dedicated to fatigue detection based on different types of signals, including eye-tracking. Section 3 includes the method description, training pipeline, and classification pipeline. Section 4 consists of the results obtained after training several algorithms. Section 5 includes discussion of our results and future research questions.

## 2. Related Work

According to a large-scale review of solutions for detecting human mental fatigue using machine learning techniques, all existing approaches are divided into four groups: image or video analysis, biological signal monitoring, vehicle motion detection, and hybrid approaches. Approaches based on biological signals are the most convenient to use and show better results, as they give high detection accuracy and can be non-invasive and therefore easy to use. Of course, the hybrid approaches show the best effectiveness for solving such a problem [8].

A huge part of developed fatigue detection methods is dedicated to the estimation of driver’s fatigue. For this purpose, participants are placed in settings that simulate driving a car. For several hours, people perform this task while their eye movements are recorded using eye-tracking. According to the review of existing methods of fatigue detection during driving, all methods are divided into three classes: mathematical models, rule, and machine learning. Additionally, fatigue detection can be categorized based on the input data: subjective feelings, biological aspects, physiological aspects, automotive, and hybrid. The first aspect includes questionnaires, and the second includes HRV, brain activity, skin activity, and oculomotor characteristics. The physiological aspect consists of the inclusion of mouth, face, head, and eye characteristics. The automotive aspect includes the wheel rotation, driver’s position, and change of environment. Finally, the hybrid aspect includes combinations of the above methods [9].

To estimate driver fatigue, authors of the paper [10] used data of the pupil size, visual trajectory, fixation time, pupil position, and visual field information changes under driving conditions in a simulator. According to the authors, the fixation duration reflects the difficulty of acquiring and processing information, and pupil size changes can reflect the degree of mental fatigue. These parameters were tested separately from each other and in combination using the FKNN. The highest accuracy was obtained when both characteristics were used (88%). Using the fuzzy K-nearest neighbor algorithm, they obtained a performance accuracy of about 89%. Additionally, they came to the conclusion that the average fixation duration and pupil area are good indicators of driver’s fatigue. However, pupil area is affected by individual differences. The results obtained by the authors, although having high accuracy for fatigue detection, are still inferior to the algorithm based on the analysis of EEG data.

Using the same k-NN algorithm, the authors created an approach for fatigue detection based on 35 types of blinks and head movements. According to their experiments, the smallest number of *k* and a small number of selected features give the best performance of the classifier. After recording 130 h of data and training the classifier, the authors obtained performance accuracies of 84.2% for binary classification and 70.0% for multiclass classification [11].

The authors of the paper [12] aimed to develop a robust fatigue detection system using binocular consistency information as a parameter for assessing the human condition. This approach allowed them to replace occluded eyes with unoccluded eyes when the face was leant to the side. Using bidirectional CNN, ESM, PERCLOS, and blink rate, they achieved an algorithm accuracy of about 97%.

The authors of the paper [13] concentrated on the detection of mental fatigue using eye-tracking. They used neural networks for this task and proposed to choose one of two options for pre-training data: manual labeling using a scale of subjective evaluation of a performance test, or labeling based on stages of task performance or types of tasks. They estimated the fatigue state as a binary parameter: present or not. Naturally, the authors pointed out that this approach to assessing an inherently cumulative state is incorrect. In order to distinguish fatigue levels, the authors used the Toeplitz Inverse Covariance-Based Clustering (TICC) algorithm on the following parameters: blink frequency and duration, pupil diameter, and gaze coordinates. In this way, they obtained three levels of fatigue. At the first level, the study participant talked about some subjective sensations, while eye movements and the correctness of task performance remained at the same normal level. At the second level, the well-being indicators significantly deteriorated, the quality of task performance dropped, and the characteristics of eye movements changed. At the last, third level, all indicators were significantly worse than the previous level.

In another study, the TICC algorithm was used as a data classifier. The authors [14] recorded eye movements and heart rate variability in parallel. HRV was divided into 1 min samples, cleaned to exclude individual differences, and analyzed using the algorithm. Thus, the authors showed that LF, LFnu, and LF/HF increased and HFnu fell. LFnu and LF/HF reflect the activity of the sympathetic system, and LF can characterize both systems. The authors suggested that the activation of the sympathetic system indicates the occurrence of fatigue. The sensitivity of HF may depend on the specific task and may be influenced by external factors, and therefore it is not sensitive enough to mental fatigue, which is confirmed by the fact that researchers obtain different results when analyzing this parameter.

Fatigue can also be assessed by tracking changes in a human posture. For example, the authors of a paper on fatigue detection in drivers [15] recorded 18 points on the body of each participant. Based on the analysis of hand movements, they developed an algorithm with a detection accuracy of about 99%. However, this approach is not applicable to all drivers, as it covers only one driving style, i.e., when the two hands of the driver are located on the wheel.

Neiry, a company producing a wide range of BCI equipment, has developed a number of classifiers for dozens of psychophysiological states, including fatigue. The human state is analyzed on the basis of EEG data recorded using several electrodes. The manufacturers claim to measure three degrees of fatigue [16].

Videomix is a company that specializes in biometric analytics. They have developed a system of gaze direction detection that provides monitoring of the operator’s functional state, which includes physical, psychological, and emotional parameters. Thus, the authors provide timely detection of critical situations [17].

At the moment, the method of fatigue detection by analyzing head movements is not so popular. This method requires installation of cameras in the workplace (or in the driver’s car, for example). Accordingly, in a review of existing algorithms, studies on the principle of camera placement, the configuration of head detection based on facial features, and their geometric configuration are described [18].

As a result of this literature review, it can be concluded that many solutions have been developed in the field of fatigue detection. However, the high variability of approaches, the use of invasive or inconvenient sensors, and the long data collection process impair the quality of the detection task. In emergency situations, such limitations can lead to fatal consequences. Therefore, there is a need to develop methods that utilize the least amount of input data and provide real-time, highly accurate results.

## 3. Fatigue Assessment Method

This section describes a method for mental fatigue assessment based on eye movement characteristic analysis. Firstly, we empirically identify eye movement characteristics that are most relevant to a mental fatigue state (characteristics that are significant when the person is fatigued). Then, we train a ML classifier that allows the distinguishing of people in fatigue and non-fatigue states based on the identified eye movement characteristics.

### 3.1. Method Description

**Figure 1:**
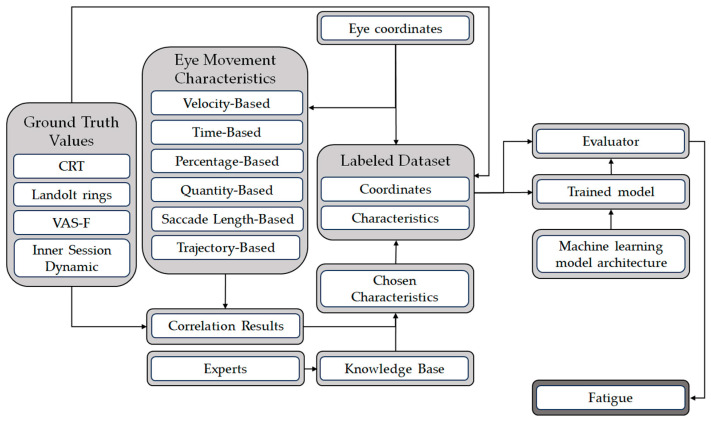
Fatigue classification pipeline.

The mental fatigue assessment method (see Figure 1) consisted of a training scenario and a fatigue assessment scenario. During the training scenario, we created a machine learning model for fatigue state classification. The result of the mental fatigue assessment scenario is an estimation of the PC operator’s fatigue state.

The training scenario starts with the calculation of eye movement characteristics from eye movement coordinates. We classified all eye movement characteristics into the following groups: velocity-based, temporal-based, percentage-based, quantitative-based, saccade length-based, and trajectory-based. Correlations between eye movement characteristics and the fatigue state were implemented based on the ground truth values available in the dataset. The ground truth values included the choice reaction time (CRT), Landolt rings test, visual analogue scale to evaluate mental fatigue severity (VAS-F), and inner session dynamic estimation. We describe these ground truth values in detail in Section 3.3. A group of experts joined together and compared correlation results and theoretical knowledge on the topic of eye movement strategies to identify chosen characteristics, as well as eye coordinates, for classifier training. We tried the following architectures: random forest, decision tree, and multilayer perceptron. We describe this process in detail in Section 3.4.

For the fatigue assessment scenario, we used the best chosen characteristics, eye coordinates, and trained classification model.

### 3.2. Dataset Description

We used our earlier developed dataset. We recorded eye-tracking data of 15 participants. For each of them, we recorded for 7 days, with each day containing three recordings of approximately one hour duration (morning session, afternoon session, and evening session). Each participant also implemented a subjective assessment of the fatigue state as the ground truth (we used Landolt rings, CRT, and VAS-F). We described the dataset in detail in our previous paper [7].

### 3.3. Detection of Eye Movement Characteristic-Related Fatigue State

This subsection aims to identify a feasible number of eye movement characteristics that are most related to the mental fatigue state by calculation of the correlations of the whole set of eye movement characteristics, with subjective assessment of the mental fatigue state.

#### 3.3.1. Landolt Rings

The Landolt rings correction test is a classic method for assessing attentional properties. The Landolt rings are emotionally neutral stimuli that are not difficult to distinguish. We proposed to use the Au index (mental performance) as a fatigue indicator. The values of this parameter within our dataset ranged from −0.5 to 4. Thus, the Au index was categorized as either low or high based on the selected threshold of 1.5. This threshold was chosen because this value allows all data to be categorized in equal proportions. If a subject had a mental performance value of 1.5 or higher, their performance was considered high, otherwise it was considered low. Then, the eye movement characteristics of these two groups were compared with each other using the Wilcoxon criterion. This non-parametric test is used for data that may not be normally distributed for dependent samples as well as for small sample sizes. The Wilcoxon signed-rank test on high and low groups provided a *p*-value of less than 0.05.

#### 3.3.2. Inner Session Dynamics

In accordance with our dataset, each session of the experiment consisted of several tasks with a 1 h duration. Each session began and ended with the CRT task. Therefore, if a characteristic was affected by fatigue, then its values would differ during these tasks, and in different sessions these differences would have the same trend. For example, in the eye movement characteristics, the proportion of fixations more than 150 ms tended to increase (this was typical for 61% of sessions in our dataset), while the value of the average curvature fell in 19% of sessions for all participants. Thus, each eye movement characteristic was assigned the highest of two values that characterize the behavior of this characteristic in cases of fatigue: (1) the proportion of sessions where it tended to increase; (2) the proportion of sessions in which it decreased.

#### 3.3.3. Choice Reaction Time (CRT)

The results of the CRT task are the values of the average reaction time, standard deviation, and number of errors. Participants completed this task at the beginning and at the end of the session. The CRT results corresponded to the values of the eye movement characteristics recorded during the session. We sorted the data based on the average reaction time and standard deviation. Then, the difference (delta) between the values of the average reaction time at the beginning and at the end of each session was calculated. Correlations were calculated between the values of the first characteristic and the corresponding delta values for the fixation area with a diameter from 0.1 to 2.5 degrees of the visual angle. The maximum correlation values of each characteristic were selected.

#### 3.3.4. Visual Analogue Scale (VAS-F)

The results of the VAS-F to evaluate mental fatigue severity are numerical scores of fatigue and energy. This test was performed once before each session. One fatigue value of the VAS-F test corresponded to the eye movement characteristic values recorded during the session. Correlations were calculated between the values of the first characteristic and the corresponding fatigue values of the VAS-F test for a fixation area with a diameter from 0.1 to 2.5 degrees of the visual angle. The maximum correlation values of each characteristic were selected. Different characteristics had maximum correlation values with different diameters of the fixation area.

#### 3.3.5. Correlation Analysis

To identify the characteristics that are the most related to mental fatigue state detection, we compared the correlation results that were calculated on the data from our dataset. In Table 1, we show the *p*-value for each characteristic in the column Landolt Rings (see Section 3.3.1). Then, we calculated the correlation analysis for each characteristic with such values as the inner session dynamics (see Section 3.3.2), CRT (see Section 3.3.3), and VAS-F (see Section 3.3.4).

We analyzed the values in the following way. Firstly, we filtered characteristics with *p*-values less than 0.05, taking them into account. Then, we sorted the values by *p*-value and involved two experts to analyze the correlations with the inner session dynamics, CRT, and VAS-F. The inner session dynamics show how the persons’ state has changed from the beginning of the session until the end, but the CRT and VAS-F show how the fatigue state has changed from session to session. Finally, we chose seven characteristics that, from the point of view of these two experts, are the most related to the fatigue state. Six characteristics were chosen as the most important from Table 1: (1) average velocity within the fixation area; (2) average curvature of the gaze trajectory; (3) minimum curvature of the gaze trajectory; (4) minimum saccade length; (5) percentage of fixations shorter than 150 ms; and (6) proportion of time spent in fixations shorter than 150 milliseconds. Additionally, the experts added one characteristic, “average speed in the fixation area”, that does not show good correlation in Table 1 but theoretically has to show correlation with human fatigue.

### 3.4. Fatigue Classification

This subsection presents the mental fatigue classification pipeline we proposed to distinguish fatigue and non-fatigue human states based on eye movement characteristics and eye coordinates.

#### 3.4.1. Data Preprocessing

**Figure 2:**
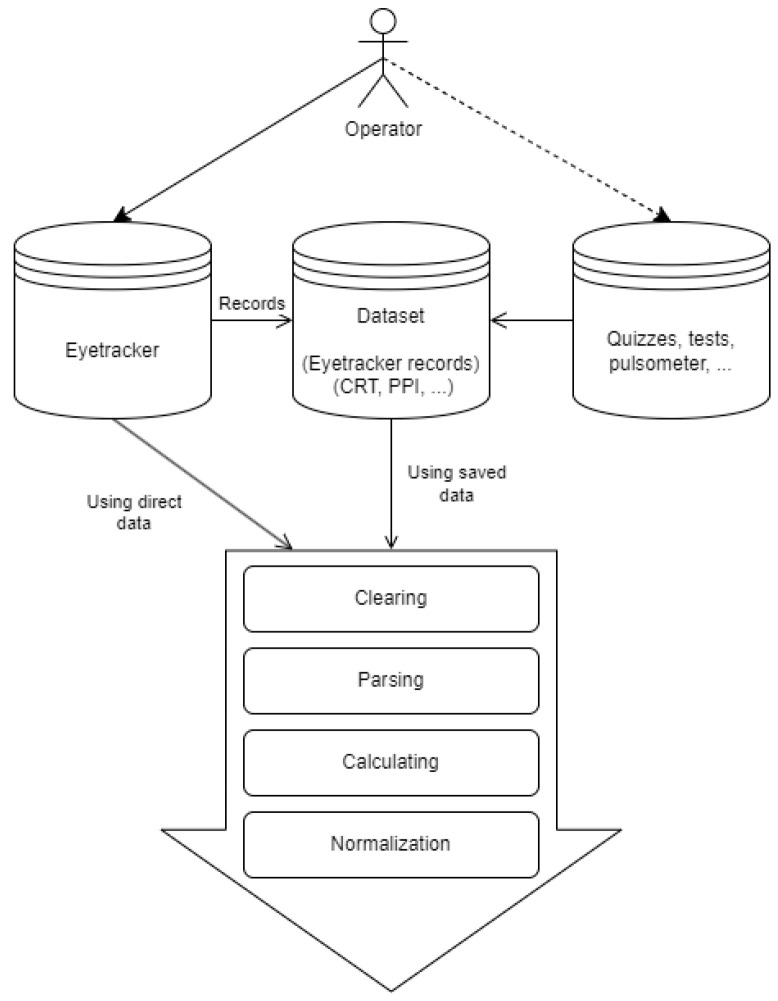
Data preprocessing.

We present the data preprocessing pipeline in Figure 2. We proposed to implement the following functions on the eye-tracking data: cleaning, parsing, calculation, and normalization. We proposed to use a cleaning function for data prefiltering, removing errors and omissions before training the model. The parsing function implements data labelling and preparation for processing. The calculation function implements gaze characteristic calculation as well as features for training the model. The normalization function implements a standard normalization procedure: mean subtraction as well as division by the mean. It is necessary to bring the various features to a near-zero neighborhood of values, which facilitates the selection of optimal weights for features in machine learning models.

The data preprocessing included three separate steps. In the initial stage, relevant features were chosen, which were represented as separate time series for each activity (set of eye movement coordinates). We computed seven key values from each feature, namely the mean, standard deviation, minimum, maximum, 25th percentile, 50th percentile, and 75th percentile. Then we prepared all characteristics presented in Table 1 (that had a *p*-value less than or equal to 0.05 using Wilcoxon). Finally, we took the seven characteristics, selected by the experts, that are the most relevant to the mental fatigue state.

#### 3.4.2. Mental Fatigue Classification Pipeline

We analyzed the ground truth data and decided that the most relevant for classification was the Landolt rings test, and we chose the mental performance (Au) parameter. If a subject had an Au value of 1.5 or higher, their performance was considered high; otherwise, it was considered low. Next, we evaluated multiple types of classifiers to determine the optimal one for our analysis. The classifiers assessed included random forest, decision tree, K-nearest neighbors, multilayer perceptron, logistic regression, and Support Vector Machine (SVM). Our dataset consisted of 1112 samples, which were divided into 912 samples for training and 200 samples for testing. The testing set comprised 100 samples with a low value of mental performance and 100 samples with a high value of mental performance. These sample sizes were chosen to maximize the amount of data available for training without compromising the reliability of the testing results.

For each classifier, we evaluated several parameter configurations to identify the optimal classifier from the available options. The goal was to select the configuration that yielded the best performance based on our evaluation criteria. The specific details of the parameter configurations and the evaluation results can be found in Table 2.

In terms of the evaluation criteria and loss functions utilized, various criteria were used during the training of the random forest and decision tree classifiers (which can be found in the previous table). Furthermore, the loss functions for the multilayer perceptron (MLP) and logistic regression classifiers were log-loss or cross-entropy loss, while for the SVM classifier we used hinge as a loss function. In contrast, the k-NN algorithm does not have a loss function that can be minimized during training and is not trained in the conventional sense. Instead, the algorithm stores a copy of the data and utilizes it during prediction. Accordingly, there is no function that is fit to the data, nor is any optimization performed.

In our study, we aimed to select the best classifier based on the highest accuracy achieved. To improve the performance of the classifiers, we applied normalization to each feature. This involved subtracting the mean from each feature and dividing the result by the standard deviation.

Additionally, we explored various feature selection methods to further enhance the classifiers’ performance. These methods included the following:

- Removing quasi-constant features (we eliminated features that had almost constant values, as they do not contribute significantly to the classification task);
- Removing correlated features (we utilized the Kendall correlation coefficient to identify and remove features that exhibited high correlation with each other);
- Moreover, we employed principal component analysis (PCA) to reduce the dimensionality of the feature space (PCA is known for its effectiveness in avoiding overfitting).

In our study, we conducted experiments using different sets or combinations of groups to investigate the impact of specific feature groups on the classification performance. The objective was to determine whether using a particular group alone or combining multiple groups would yield better results. The main indicator of the quality of the performed estimation was the F1-score, which is a combination of the accuracy and completeness of the estimates given by the classifier.

## 4. Results

**Figure 3:**
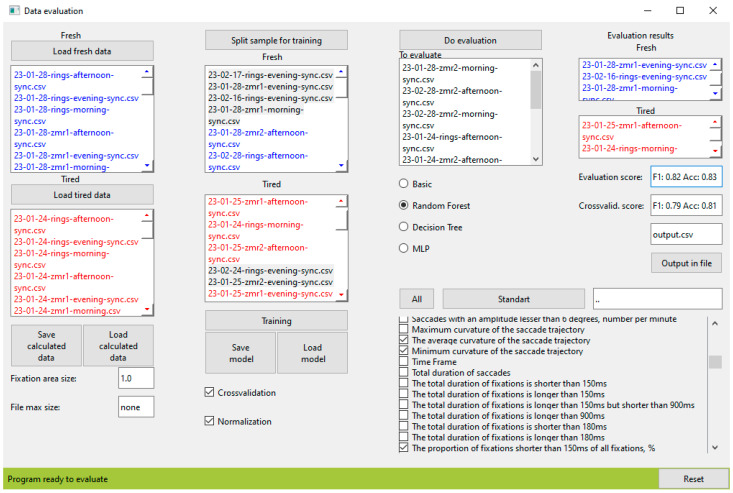
FAEyeTON system interface.

We have developed and published an open-source Python library, now available both at the GitHub and PyPI repositories, under the names FAEyeTON and eyetrackfatigue, respectively. The library is a ready-to-use software; it can be integrated into a complex system, or further modified, depending on the user’s requirements. The library is divided into several software modules that can be used separately for their main functions: reading raw data from an eye-tracker device; parsing the data and calculating features; and training and using fatigue evaluation models. Examples of such usage, in the form of test files, are available on the GitHub repository, along with supporting documentation. We developed an interface to process data that used the mentioned modules (see Figure 3).

We conducted experiments using the developed library that implemented the proposed Section 3 method. In Table 3, we present the most relevant results for the three best algorithms, random forest, decision tree, and MLP, as well as the results of experiments with the characteristics set (coordinates, selected characteristics by experts, and all characteristics presented in Table 3) that we discussed in Section 3.4.2. The goal was to determine whether using a single set or a combination of several sets would produce better results. We experimented with a random dataset, as well as implemented 10-fold cross validation that increased the trustworthiness of our results. As we can see from Table 3, the most accurate classifier is based on the random forest architecture and uses eye movement coordinates as input data together with the characteristics selected by the experts. The best accuracy after cross validation is 0.85, and the F1-score is 0.80. Therefore, we can conclude that the presented method and selected characteristics show the best results for the mental fatigue classification task.

## 5. Conclusions

This paper proposed a developed method for mental fatigue assessment based on eye movement analysis. In the scope of this method, we proposed a technique for selecting the best relevant eye movement characteristics that are related to the mental fatigue state. We identified these characteristics for our dataset and developed a classifier that shows that, using the selected characteristics, we achieved the best accuracy and F1-score for the task of mental fatigue classification (we tested and presented results for several best architectures: random forest, decision tree, and multilayered perceptron). According to the results of our experiments, using our method, we obtained an accuracy of up to 0.85 percent and an F1-score of 0.80 after implementing 10-fold cross validation. We developed an open-source library that implements the proposed method and allows users to utilize the proposed classifier as well as train their own classifier using their own data. The library can be easily integrated into target business software that needs to assess human mental fatigue in real time based on eye movements. The library spends 0.15 s to calculate the eye movement characteristics and less than 0.01 s to operate the classifier to process one second of data.

## Figures and Tables

**Table 1:** Methods of fatigue detection.

Characteristics	Landolt Rings		Inner Session Dynamics		CRT	VAS-F
Average velocity within the fixation area, °/s	0.00	0.00	−86.61%	−58.20%	10.74%	27.84%
Average curvature of the gaze trajectory	0.00	0.00	72.62%	63.91%	−9.07%	−27.34%
Min. velocity of gaze movement in the second interval, °/s	0.00	0.00	−69.05%	−49.34%	11.15%	15.99%
Minimum velocity within the fixation area, °/s	0.00	0.00	−66.52%	−45.58%	5.73%	9.17%
Minimum curvature of the gaze trajectory	0.00	0.00	56.70%	38.27%	27.34%	−22.44%
Minimum saccade length, °	0.00	0.00	56.35%	38.59%	9.76%	−40.39%
Maximum saccade length, °	0.00	0.00	−55.90%	−15.79%	8,83%	−6.53%
Percentage of fixations between 150 and 900 ms, %	0.00	0.00	55.84%	15.70%	−11.58%	12.17%
Average saccade length, °	0.00	0.01	52.27%	21.34%	−21.73%	8.35%
Average saccade duration, s	0.00	0.01	52.27%	14.27%	−21.93%	13.40%
Total duration of fixations between 150 and 900 ms, s	0.00	0.02	−21.43%	−10.12%	19.21%	8.59%
Proportion of time spent in fixations between 150 and 900 ms, %	0.00	0.01	−12.01%	−8.73%	−0.50%	28.57%
Maximum instantaneous gaze velocity, °/s	0.01	0.00	−55.90%	−23.90%	−0.50%	1.18%
Maximum curvature of the gaze trajectory	0.01	0.02	−2.78%	−8.08%	−18.64%	−8.28%
Percentage of fixations shorter than 150 ms, %	0.01	0.05	−59.82%	−31.28%	16.20%	7.16%
Percentage of fixations longer than 150 ms, %	0.01	0.05	59.82%	40.73%	−16.53%	−19.05%
Percentage of fixations shorter than 180 ms, %	0.01	0.05	−68.06%	−29.91%	13.72%	4.93%
Percentage of fixations longer than 180 ms, %	0.01	0.05	66.07%	29.35%	−13.96%	7.09%
Average saccade velocity, °/s	0.01	0.04	−0.45%	−0.80%	−4.31%	8.32%
False fixations per minute	0.01	0.10	−62.50%	−21.41%	19.84%	12.37%
Modulus of average acceleration in second interval, °/s^2^	0.01	0.10	−73.26%	−37.74%	−5.29%	7.30%
Fixations longer than 900 ms per minute	0.02	0.10	−10.71%	−13.33%	−8.24%	14.81%
Proportion of time spent in fixations shorter than 150 milliseconds, %	0.03	0.04	−82.86%	−53.97%	23.51%	15.41%
Maximum saccade duration, s	0.04	0.13	−59.13%	−18.16%	17.46%	10.55%
Fixations shorter than 180 ms per minute	0.05	0.07	−3.57%	−11.37%	−3.60%	19.08%
Proportion of time spent in fixations longer than 150 ms, %	0.05	0.10	82.86%	50.69%	−12.52%	−10.71%

**Table 2:** Parameter configurations.

Classifier	Parameters	Attempted Values of the Parameters
Random forest	Number of estimators	[10, 20, 30, 40, 50, 60, 70, 80, 90, 100]
	Criterion	[‘gini’, ‘entropy’, ‘log_loss’]
Decision tree	Criterion	[‘gini’, ‘entropy’, ‘log_loss’]
K-nearest neighbors	Number of neighbors	[1, 2, 3, 4, 5, 6, 7, 8, 9, 10]
	Weights	[‘uniform’, ‘distance’]
MLP	Solver	[‘lbfgs’, ‘sgd’, ‘adam’]
	Activation function	[‘identity’, ‘logistic’, ‘tanh’, ‘relu’]
	Hidden layer sizes	From 1 to 20
Logistic regression	Solver	[‘lbfgs’, ‘liblinear’, ‘newton-cg’, ‘sag’, ‘saga’]
SVM	Kernel	[‘linear’, ‘poly’, ‘rbf’, ‘sigmoid’]

**Table 3:** Results of algorithm approbation.

Characteristics	Algorithm	Random Set		Cross Validation	
		Accuracy	F1-Score	Accuracy	F1-Score
Coordinates	Random forest	0.87	0.82	0.84	0.80
Selected char.	Random forest	0.87	0.83	0.84	0.80
All characteristics	Random forest	0.86	0.82	0.84	0.79
Coord + selected char.	Random forest	0.84	0.78	0.85	0.80
Coordinates	Decision tree	0.74	0.69	0.77	0.73
Selected char.	Decision tree	0.77	0.73	0.76	0.72
All characteristics	Decision tree	0.76	0.71	0.76	0.74
Coord + selected char.	Decision tree	0.74	0.71	0.77	0.73
Coordinates	MLP	0.85	0.90	0.83	0.78
Selected char.	MLP	0.83	0.80	0.83	0.78
All characteristics	MLP	0.83	0.76	0.83	0.78
Coord + selected char.	MLP	0.85	0.81	0.83	0.77

## Data Availability

The developed open-source library is available at https://github.com/AI-group-72/FAEyeTON (access date: 21 October 2024).
